# Enhanced Carbapenem Resistance through Multimerization of Plasmids Carrying Carbapenemase Genes

**DOI:** 10.1128/mBio.00186-21

**Published:** 2021-06-22

**Authors:** Ryuichiro Abe, Yukihiro Akeda, Yo Sugawara, Yuki Matsumoto, Daisuke Motooka, Ryuji Kawahara, Norihisa Yamamoto, Kazunori Tomono, Tetsuya Iida, Shigeyuki Hamada

**Affiliations:** a Japan-Thailand Research Collaboration Center on Emerging and Re-emerging Infections, Research Institute for Microbial Diseases, Osaka University, Osaka, Japan; b Department of Bacterial Infections, Research Institute for Microbial Diseases, Osaka University, Osaka, Japan; c Division of Infection Control and Prevention, Osaka University Hospital, Suita, Japan; d Department of Infection Control and Prevention, Graduate School of Medicine, Osaka University, Osaka, Japan; e National Institute of Infectious Diseases, Tokyo, Japan; f Department of Infection Metagenomics, Research Institute for Microbial Diseases, Osaka University, Osaka, Japan; g Department of Microbiology, Osaka Institute of Public Health, Osaka, Japan; Emory University; University of Oxford

**Keywords:** carbapenemase, plasmid multimerization, mechanism of antimicrobial resistance, carbapenem-resistant *Enterobacteriaceae*, *recA*

## Abstract

The worldwide dissemination of carbapenem-resistant *Enterobacteriaceae* (CRE) poses a critical human health issue by limiting the range of antibiotics that are usable in the treatment of common bacterial infections. Along with CRE, carbapenem heteroresistance has disseminated worldwide, which is described as different levels of carbapenem resistance within a seemingly isogenic bacterial population. Unstable carbapenem resistance will likely lead to unexpected treatment failure due to the enhanced resistance after initiation of treatment, contradicting antimicrobial susceptibility test results. Porin mutation and tandem amplification of the carbapenemase gene have been reported as mechanisms underlying enhanced carbapenem resistance. In this study, we identified multimerization of plasmids carrying carbapenemase genes, by using Southern blotting, whole-genome sequencing, and quantitative PCR (qPCR) analysis for the CRE isolates obtained in our previous surveillance in Osaka, Japan. Plasmids harboring a carbapenemase gene were multimerized by *recA*, likely through recombination at two consecutive sets of transposase genes of the IS*91* family, thereby producing various plasmids of discrete sizes in a single bacterial cell of an Escherichia coli isolate. This multimerization resulted in increased copy numbers of carbapenemase genes, leading to enhanced gene transcription as well as carbapenem resistance. Prior exposure to meropenem further increased the copy number of carbapenemase genes, readily resulting in enhancement of carbapenem resistance. This mechanism may lead to clinical treatment failure by sifting antimicrobial resistance after the treatment initiation.

## OBSERVATION

The rapid global dissemination of multidrug-resistant (MDR) *Enterobacteriaceae* threatens health care systems worldwide (1). Among MDR *Enterobacteriaceae*, carbapenem-resistant *Enterobacteriaceae* (CRE) are of major concern because alternative treatment options are limited even against common bacterial infections. Carbapenem resistance is conferred primarily by carbapenemases, which hydrolyze carbapenems (2, 3). The level of carbapenem resistance differs among carbapenemase-producing isolates, with some carbapenemase producers exhibiting heteroresistance to carbapenems (4). Different levels of resistance to a particular antibiotic within a seemingly isogenic bacterial population, which is described as heteroresistance (5, 6), may lead to treatment failure of infectious diseases after the initiation of antibiotic therapy (7). Notably, heteroresistance against carbapenems has already spread worldwide (8, 9). There are several systems underlying enhancement of carbapenem resistance. To date, functional mutation of porins in the bacterial cell membrane that decreases their permeability to carbapenems (10, 11) and tandem amplification of plasmid-borne carbapenemase genes (4, 12, 13) have been reported to enhance carbapenem resistance. Although resistance-nodulation-division efflux systems extrude a wide variety of substrates, including different classes of antibiotics (14), the role of efflux pumps in expelling carbapenems in *Enterobacteriaceae* is controversial (15). Further analysis of mechanisms for enhanced carbapenem resistance is needed to investigate the associated mechanisms for heteroresistance. Thus, in this study, we report a novel mechanism for the enhancement of carbapenem resistance.

**FIG 1:**
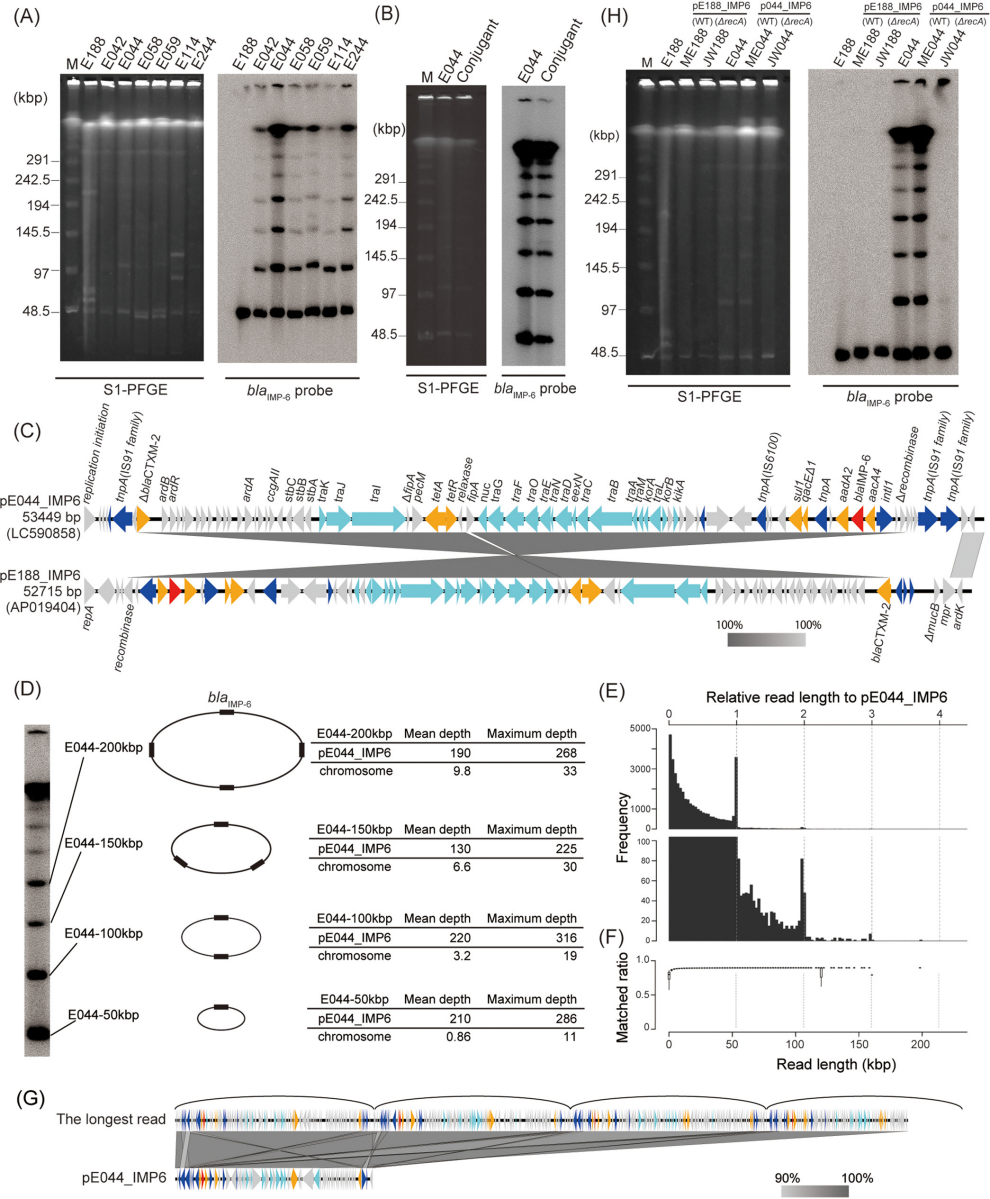
Multimerization of plasmids carrying *bla*_IMP-6_ in E. coli. (A) Ladder bands on a Southern blot using a *bla*_IMP-6_ probe following S1-PFGE. Southern blotting with a *bla*_IMP-6_ probe following PFGE of S1-digested genomic DNA from E. coli isolates E042, E044, E058, E059, E114, and E244 revealed ladder patterns, whereas that of K. pneumoniae isolate E188 carrying a plasmid with *bla*_IMP-6_ (pE188_IMP6) revealed a single band. Lane M, DNA size marker (lambda ladder; Bio-Rad). (B) Ladder band pattern of the wild type and transconjugant. The ladder band on a Southern blot with a *bla*_IMP-6_ probe following S1-PFGE in isolate E044 was detected in a pE044_IMP6 conjugant E. coli isolate as well. (C) Comparison of plasmids pE044_IMP6 and pE188_IMP6. pE044_IMP6 consisted of pE188_IMP6 juxtaposed with three sets of transposase genes of the IS*91* family. Block arrows indicate confirmed or putative open reading frames and their orientations. Arrow size is proportional to the predicted open reading frame length. The color code is as follows: red, carbapenem resistance gene; yellow, other antimicrobial resistance gene; light blue, conjugative transfer gene; blue, mobile element. Putative, hypothetical, or unknown genes are represented by gray arrows. (D) DNA fragments contained in each ladder band included only the sequence of pE044_IMP6. DNA fragments extracted from bands at 50, 100, 150, and 200 kbp by S1-PFGE were sequenced by an Illumina MiSeq system. Mean and maximum depths of sequence reads mapped against pE044_IMP6 and the full-length chromosome indicated that each band consisted of multimerized plasmid pE044_IMP6. (E) Size distribution of plasmid reads obtained from ultra-long-read sequencing. The top and bottom of the plots are drawn in different *y*-axis value ranges. The frequencies of the integer multiplication of relative length were higher than surrounding ones. (F) Box plot in each bin of size distribution. The matched ratio was calculated as the ratio of the number of matched nucleotides to the read length. (G) Comparison of the genomic structures of the longest sequence read and plasmid pE044_IMP6. The longest read obtained from ultra-long-read sequencing was comprised of almost four multimerized monomer plasmids. (H) pE044_IMP6 multimerized by *recA*. Southern blotting with a *bla*_IMP-6_ probe following S1-PFGE was conducted for E. coli isolates E188 and E044, pE188_IMP6 transformants ME188 and JW188 (*recA*-deficient ME188), and pE044_IMP6 transformants ME044 and JW044 (*recA*-deficient ME044).

Among the CRE isolates obtained in our previous surveillance (4), we identified six chromosomally distinct Escherichia coli isolates (E042, E044, E058, E059, E114, and E244) that exhibited similar ladder patterns on Southern blot hybridization by use of a *bla*_IMP-6_ probe following S1 nuclease-digested pulsed-field gel electrophoresis (S1-PFGE) analysis (Fig. 1A). The ladder pattern bands were preserved even after the plasmids were conjugated into another E. coli strain, TUM3456 (Fig. 1B). However, whole-genome sequencing analysis of E. coli isolate E044 using a Nanopore GridION (Oxford Nanopore Technologies, UK) after treatment with an SQK-LSK109 1D ligation sequencing kit indicated that it carried only one IncN plasmid, pE044_IMP6, with a size of 53,449 bp; this plasmid exhibited 99.99% identity to and 93% coverage of plasmid pE188_IMP6 (Fig. 1C), which showed a single band by Southern blotting following S1-PFGE. pE188_IMP6 is a 52,715-bp IncN plasmid obtained from Klebsiella pneumoniae isolate E188 in our previous surveillance as a predominant *bla*_IMP-6_-carrying plasmid spreading in Osaka, Japan (4). In the sequence of pE044_IMP6, pE188_IMP6 was bracketed with three sets of transposase genes of the IS*91* family. Southern blotting profiles indicated that these isolates carried multiple plasmids harboring *bla*_IMP-6_, and the plasmid sizes presented an arithmetic progression (Fig. 1A). The smallest plasmid was approximately 50 kbp, which was equal to the common difference of arithmetic progression, implying that these plasmids are multimers of a 50-kbp plasmid. The discrete sizes of the bands, even after treatment with proteinase K and nuclease S1, suggested that these bands did not represent an accumulation of monomers but that each band represented a single, circular multimer. Next, we compared the sequences of DNA fractions contained in each S1-PFGE band with the whole genome of isolate E044 (Fig. 1D). Each ladder band plasmid consisted of the DNA sequence of pE044_IMP6, without any other sequence of the whole chromosomal sequence of isolate E044. These results indicated that each S1-PFGE band represented multimerization of the 53-kbp plasmid, pE044_IMP6. The uniformity in the depth of reads from each band mapped on the pE044_IMP6 sequence indicated the absence of replicative amplification of insertion sequences, thereby implying that the plasmids were multimerized by homologous recombination and not by replicative transposition of insertion sequences (see Fig. S1 in the supplemental material) (16). Additionally, whole-genome sequencing analysis of isolate E044 using MinION for ultra-long-read sequencing confirmed the existence of sequence reads exhibiting plasmid multimers (Fig. 1E and F). The longest read was compared with pE044_IMP6, indicating multimerization of almost four monomer plasmids despite the low resolution (Fig. 1G; Fig. S2). All the monomer plasmids connected unidirectionally to form multimers without any monomer plasmid connected in the opposite direction (Fig. S2). This isolate exhibits multimerization of the clinically prevalent plasmid, which is considerably larger than the multimers of small plasmids reported to date (17–19).

10.1128/mBio.00186-21.1 FIG S1 Depth of reads mapped on pE044_IMP6. DNA fragments extracted from bands with sizes of 50 kbp (A), 100 kbp (B), 150 kbp (C), and 200 kbp (D) in S1-PFGE were sequenced using an Illumina MiSeq. The read depth against pE044_IMP6 is indicated. The three blue shades represent minimum, average, and maximum coverage values for the aggregated mapped reads. Download FIG S1, PDF file, 0.5 MB. Copyright © 2021 Abe et al. 2021 Abe et al. https://creativecommons.org/licenses/by/4.0/ This content is distributed under the terms of the Creative Commons Attribution 4.0 International license.

10.1128/mBio.00186-21.2 FIG S2 Plasmid multimerization observed by MinION. The longest read (198 kb) obtained from ultra-long-read sequencing of isolate E044 was mapped 3.7 times serially to pE044_IMP6 (53 kb) in the forward strand. The colors of the lines show strand direction of the longest read (198 kb) compared with pE044_IMP6, as forward (red) and reverse (blue). Download FIG S2, PDF file, 0.5 MB. Copyright © 2021 Abe et al. 2021 Abe et al. https://creativecommons.org/licenses/by/4.0/ This content is distributed under the terms of the Creative Commons Attribution 4.0 International license.

Plasmid in isolate E044 was conjugated into E. coli strain ME9062 and its isogenic *recA* mutant, designated strains ME044 and JW044, respectively. Southern blotting hybridization using a *bla*_IMP-6_ probe following S1-PFGE analysis showed the disappearance of ladder pattern bands in the *recA*-deficient strain, JW044 (Fig. 1H). Plasmid in isolate JW044 was further conjugated into E. coli strain TUM3456 carrying wild-type *recA*, demonstrating the reproducibility of ladder pattern bands in the isolate (Fig. S3). We demonstrated that *recA* mediated the multimerization of the plasmids, likely through homologous recombination (17, 20). The difference between pE188_IMP6 and pE044_IMP6 was the presence of several copies of transposases suspected as the origin of multimerization. A comparison of the sequences of plasmids pE044_IMP6 and pJW044, which should be identical, further suggested that the recombination targeting two consecutive sets of transposase genes of the IS*91* family caused the difference between the structures of these plasmids (Fig. S4).

10.1128/mBio.00186-21.3 FIG S3 Reproducibility of multimer patterns. The ladder band observed by Southern blotting with a *bla*_IMP-6_ probe following S1-PFGE in isolate E044 changed into a single band after conjugation into *recA*-negative E. coli isolate JW044. The plasmid in JW044 was further conjugated into E. coli isolate TUM3456 with wild-type *recA*, indicated as the TUM-JW044 transconjugant. The ladder band was observed by Southern blotting with a *bla*_IMP-6_ probe following S1-PFGE in the TUM-JW044 transconjugant. Download FIG S3, PDF file, 0.8 MB. Copyright © 2021 Abe et al. 2021 Abe et al. https://creativecommons.org/licenses/by/4.0/ This content is distributed under the terms of the Creative Commons Attribution 4.0 International license.

10.1128/mBio.00186-21.4 FIG S4 Comparison of the genome structures of plasmids pE044_IMP6 and pJW044. Plasmid pJW044 in *recA*-deficient transformant JW044, which was not multimerized, was compared with the original plasmid, pE044_IMP6. The consecutive transposases likely targeted for homologous recombination leading to multimerization are indicated by arrows. Block arrows indicate confirmed or putative open reading frames and their orientations. Arrow size is proportional to the predicted open reading frame length. The color code is as follows: red, carbapenem resistance gene; yellow, other antimicrobial resistance gene; light blue, conjugative transfer gene; blue, mobile element. Putative, hypothetical, or unknown genes are represented by gray arrows. Download FIG S4, PDF file, 0.5 MB. Copyright © 2021 Abe et al. 2021 Abe et al. https://creativecommons.org/licenses/by/4.0/ This content is distributed under the terms of the Creative Commons Attribution 4.0 International license.

**FIG 2:**
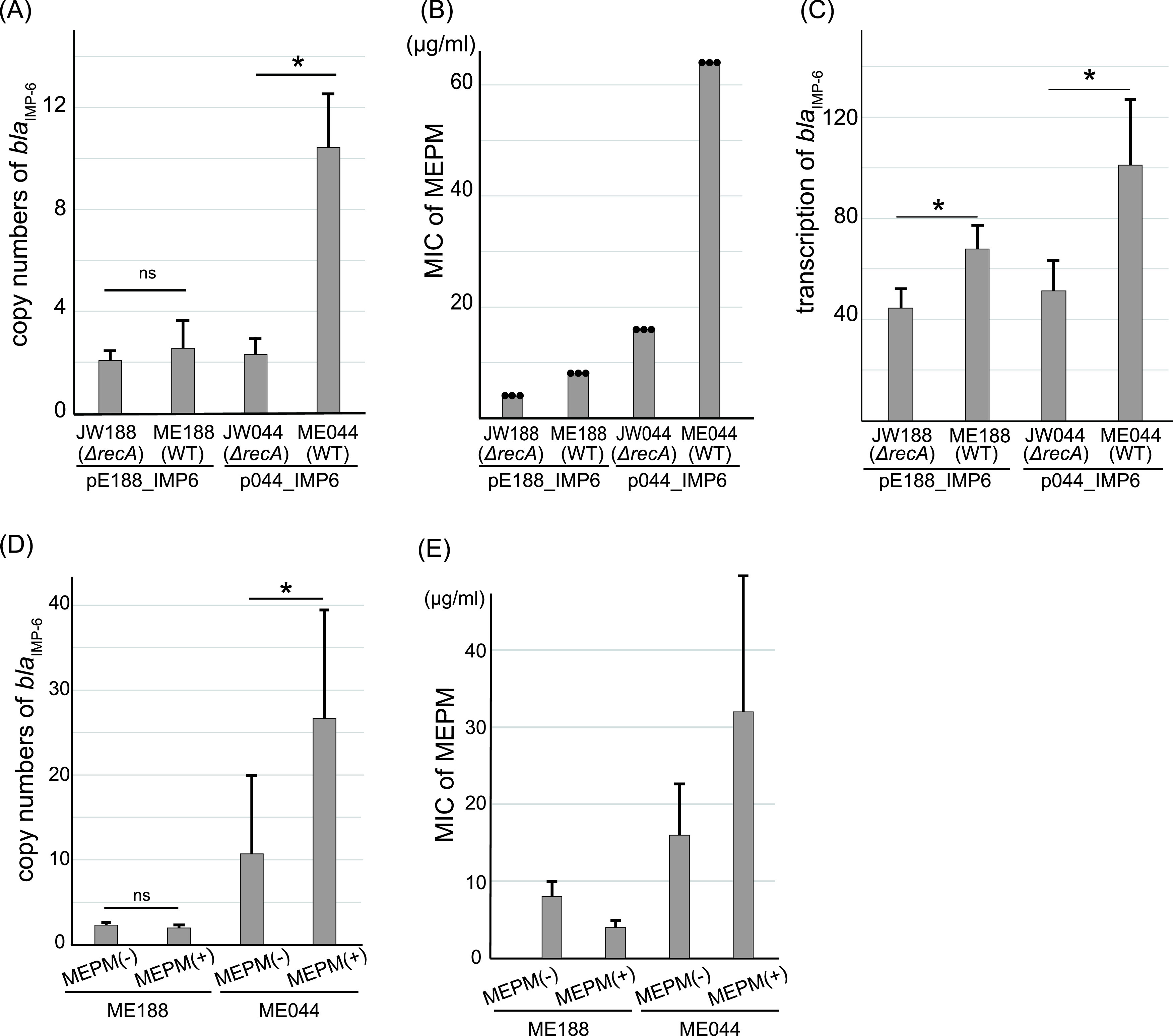
Plasmid multimerization enhances carbapenem resistance. (A) Copy numbers of *bla*_IMP-6_ per cell of E. coli transformants JW188, ME188, JW044, and ME044. Copy numbers of *bla*_IMP-6_ in transformant cells were determined by qPCR, using *rrsA* as an internal control gene. Bars indicate the mean ± standard deviation, calculated from quadruple experiments. WT; wild type. (B) MICs of meropenem (MEPM) for E. coli transformants. Points indicate the results of triplicate experiments. (C) Transcript levels of *bla*_IMP-6_ in E. coli transformants. *bla*_IMP-6_ transcription in transformant cells was measured by reverse transcription (RT)-qPCR. The bar chart represents the relative mRNA transcript ratio of *bla*_IMP-6_ to that of *rrsA*. Bars indicate the mean ± standard deviation calculated from quintuple experiments. (D) Increased *bla*_IMP-6_ copy numbers in ME044 cells after overnight exposure to meropenem. Copy numbers of *bla*_IMP-6_ in transformant cells were determined by qPCR, using *rrsA* as an internal control. Bars indicate the mean ± standard deviation, calculated from nonuple experiments. (E) The MIC of meropenem for transformant ME044 is increased after preexposure (overnight) to meropenem. MICs were measured by modified methods (see Text S1 in the supplemental material). The bars indicate medians ± standard deviation calculated from nonuple experiments. Statistical analysis was performed using Mann-Whitney U tests; *, *P < *0.05; ns, not significant.

We then investigated the effect of plasmid multimerization on antimicrobial resistance. Quantitative PCR (qPCR) revealed that strain ME044 cells harbored an increased copy number of *bla*_IMP-6_ when compared with strain JW044, whereas the copy number of *bla*_IMP-6_ in strain ME188 was not significantly different from that of JW188 (Fig. 2A). In addition, *bla*_IMP-6_ transcription and the MIC of meropenem were significantly higher for strain ME044 than for JW044 (Fig. 2B and C). These results indicated that the plasmid multimerization led to increased *bla*_IMP-6_ copy numbers and transcription in the bacterial cells, resulting in enhanced meropenem resistance. The presence of *recA* probably affects enhancement of meropenem resistance in ME188 or ME044 (21). However, the exceptional resistance to meropenem in ME044 compared with that of other transformants should be induced by increased *bla*_IMP-6_ transcription following plasmid multimerization.

10.1128/mBio.00186-21.7 TEXT S1 Detailed materials and methods of techniques used in this work. Download Text S1, DOCX file, 0.04 MB. Copyright © 2021 Abe et al. 2021 Abe et al. https://creativecommons.org/licenses/by/4.0/ This content is distributed under the terms of the Creative Commons Attribution 4.0 International license.

Interestingly, preexposure to meropenem (overnight) increased the copy number of *bla*_IMP-6_ in ME044 cells (Fig. S5 and Fig. 2D), but it did not affect the copy number in ME188. This additional increase in *bla*_IMP-6_ copy number further enhanced the meropenem resistance of ME044 (Fig. 2E). Figure S6 shows the emergence of colonies within the inhibition zone of ME044, which exhibited higher resistance against meropenem after meropenem exposure. Exposure to meropenem is considered a burden on ME044 cells, increasing *recA* activity and subsequently inducing homologous recombination along with genome repair (22). These processes may cause increased plasmid multimerization, leading to increased *bla*_IMP-6_ copy numbers and enhanced meropenem resistance. However, increased *bla*_IMP-6_ copy number could also be due to the selection of the subpopulation carrying a higher number of multimers from a population that should be isogenic. Our results demonstrated the heterogeneity of *bla*_IMP-6_ copy numbers within seemingly isogenic clones, and meropenem exposure enhanced the resistance of the population through the increased copy number of *bla*_IMP-6_ in the population. Further analysis comparing maximum *bla*_IMP-6_ copy numbers in single cells should be conducted to confirm the promotion of multimerization via meropenem stimulation.

10.1128/mBio.00186-21.5 FIG S5 Schematic procedure of meropenem preexposure. Download FIG S5, PDF file, 0.5 MB. Copyright © 2021 Abe et al. 2021 Abe et al. https://creativecommons.org/licenses/by/4.0/ This content is distributed under the terms of the Creative Commons Attribution 4.0 International license.

10.1128/mBio.00186-21.6 FIG S6 Emergence of enhanced meropenem-resistant clone through meropenem exposure. Meropenem MICs of isolates ME188 and ME044 with or without meropenem exposure were measured by Etest. Download FIG S6, PDF file, 0.5 MB. Copyright © 2021 Abe et al. 2021 Abe et al. https://creativecommons.org/licenses/by/4.0/ This content is distributed under the terms of the Creative Commons Attribution 4.0 International license.

In conclusion, carbapenemase-encoding plasmids formed multimers within a bacterial cell of CRE isolates through *recA* function. This plasmid multimerization led to increased copy numbers of carbapenemase genes and enhancement of carbapenem resistance, and meropenem exposure resulted in a further increase in carbapenemase genes and enhanced resistance. This mechanism may underlie enhanced resistance to other antimicrobials and may likely cause eventual treatment failure of infectious diseases that initially respond to the treatment with antibiotics.

### Data availability.

The whole-genome sequencing data are available from the DNA Data Bank of Japan (DDBJ) under accession numbers AP019404, LC590858, and LC594662. Raw sequence data of DNA fractions extracted from ladder band gels are available at NCBI under accession numbers DRX229219 to DRX229222. Raw data of E044 sequenced by MinION for ultra-long-read sequencing is available at NCBI under accession number DRX264853.
