# Supplementary material for: Enhanced Carbapenem Resistance through Multimerization of Plasmids Carrying Carbapenemase Genes
Source: mBio. 2021 Jun 22;12(3):e00186-21. doi: 10.1128/mBio.00186-21 (PMC8262910; doi:10.1128/mBio.00186-21)
Supplement: FIG S1 [file mbio.00186-21-sf001.pdf]

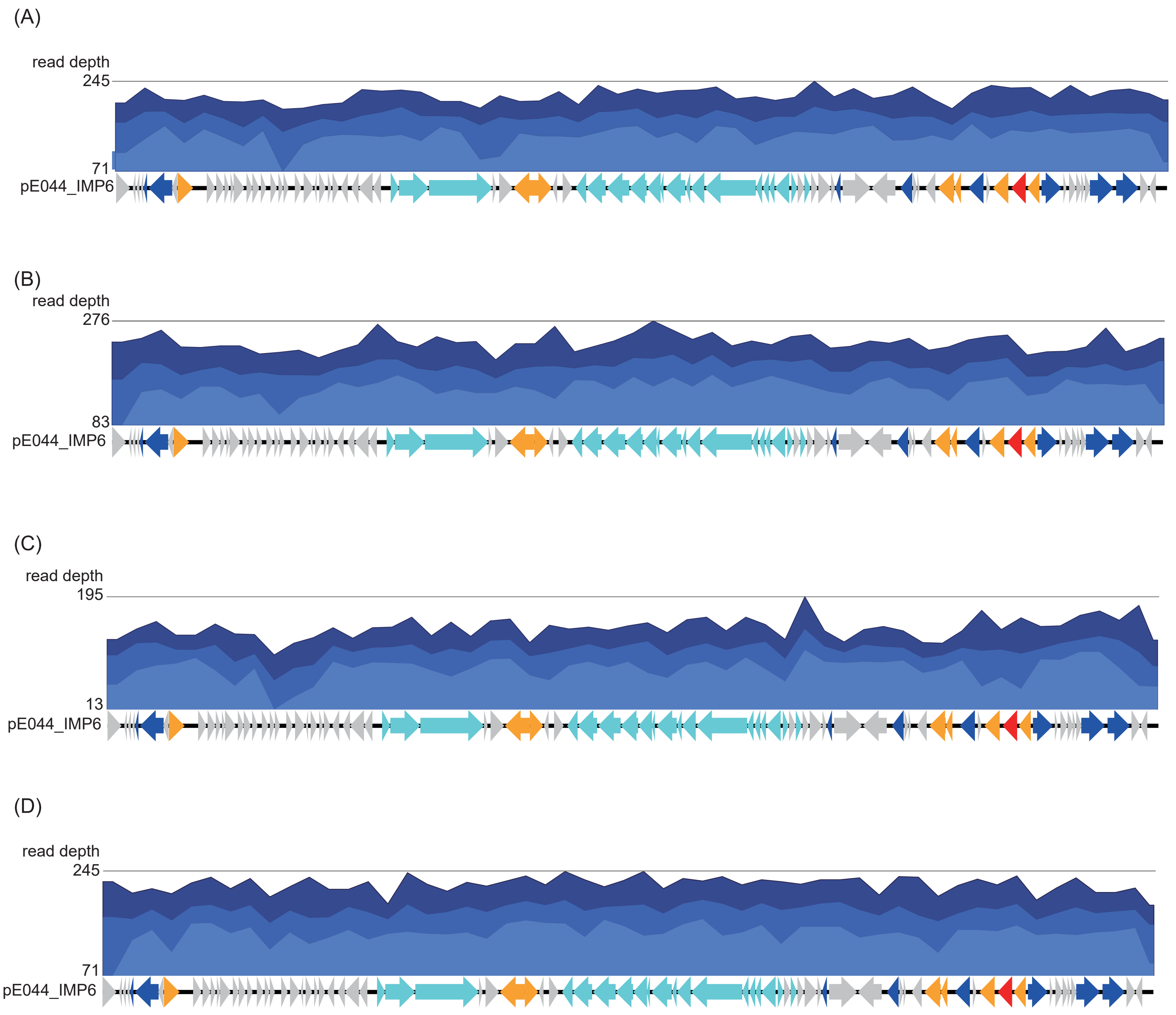


**FIG S1 Depth of reads mapped on pE044_IMP6.** DNA fragments extracted from bands with sizes of (A) 50 kbp, (B) 100 kbp, (C) 150 kbp, and (D) 200 kbp in S1-PFGE were sequenced by Illumina MiSeq. The read depth against pE044_IMP6 is indicated. The three blue shades represent minimum, average and maximum coverage values for the aggregated mapped reads.
