# Supplementary material for: Enhanced Carbapenem Resistance through Multimerization of Plasmids Carrying Carbapenemase Genes
Source: mBio. 2021 Jun 22;12(3):e00186-21. doi: 10.1128/mBio.00186-21 (PMC8262910; doi:10.1128/mBio.00186-21)
Supplement: FIG S2 [file mbio.00186-21-sf002.pdf]

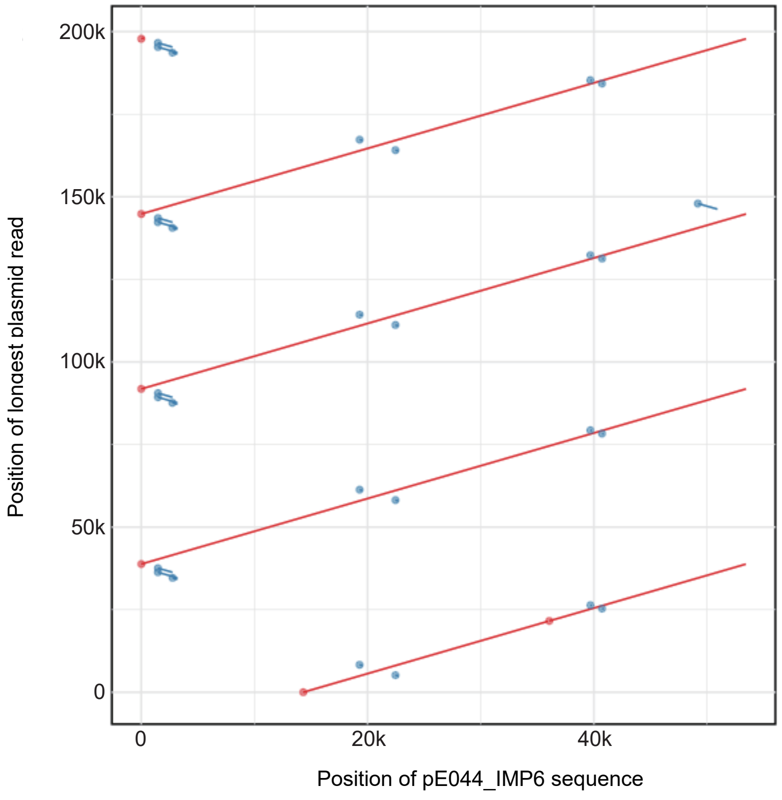

FIG S2. Plasmid multimerization observed by MinION. The longest read (198 kb) obtained from ultra-long sequencing of isolate E044 was mapped 3.7 times serially to pE044_IMP6 (53 kb) in the forward strand. The colors of the lines show strand direction of the longest read (198 kb) compared with pE044_IMP6, as forward (red) and reverse (blue).
