# Supplementary material for: Enhanced Carbapenem Resistance through Multimerization of Plasmids Carrying Carbapenemase Genes
Source: mBio. 2021 Jun 22;12(3):e00186-21. doi: 10.1128/mBio.00186-21 (PMC8262910; doi:10.1128/mBio.00186-21)
Supplement: FIG S3 [file mbio.00186-21-sf003.pdf]

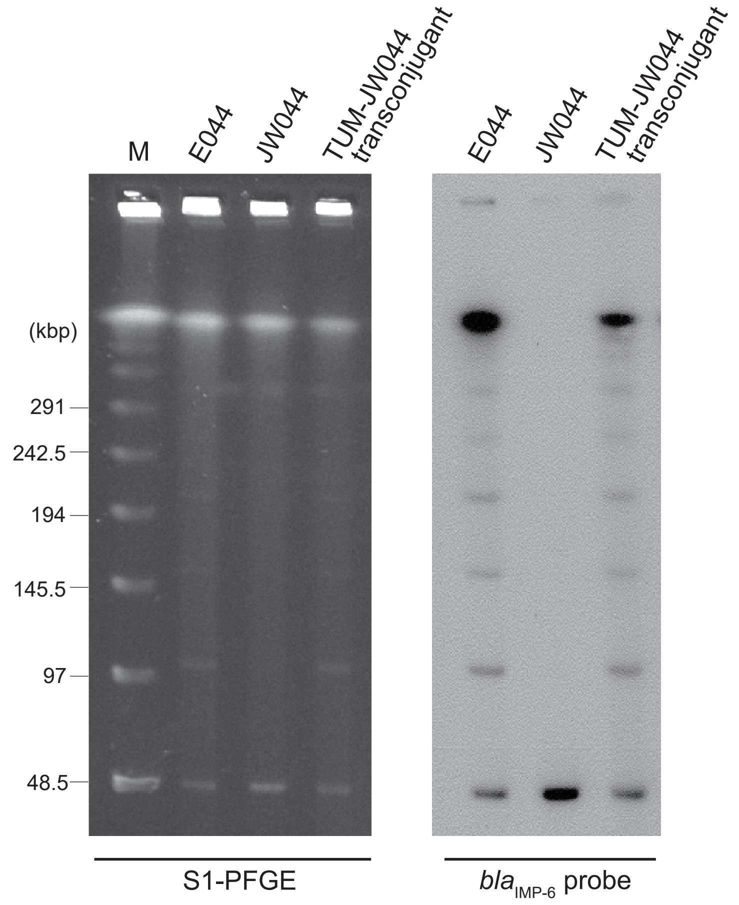

FIG S3 Reproducibility of multimer patterns. The ladder band on Southern blotting with *bla*$_{IMP-6}$ probe following S1-PFGE in isolate E044 changed into single band after conjugation into *recA*-negative *E. coli* isolate JW044. The plasmid in JW044 was further conjugated into TUM3456 *E. coli* isolate with wildtype *recA*, indicated as TUM-JW044 transconjugant.
The ladder band was observed on Southern blotting hybridization with *bla*$_{IMP-6}$ probe following S1-PFGE in TUM-JW044 transconjugant.
