# Supplementary material for: Enhanced Carbapenem Resistance through Multimerization of Plasmids Carrying Carbapenemase Genes
Source: mBio. 2021 Jun 22;12(3):e00186-21. doi: 10.1128/mBio.00186-21 (PMC8262910; doi:10.1128/mBio.00186-21)
Supplement: FIG S4 [file mbio.00186-21-sf004.pdf]

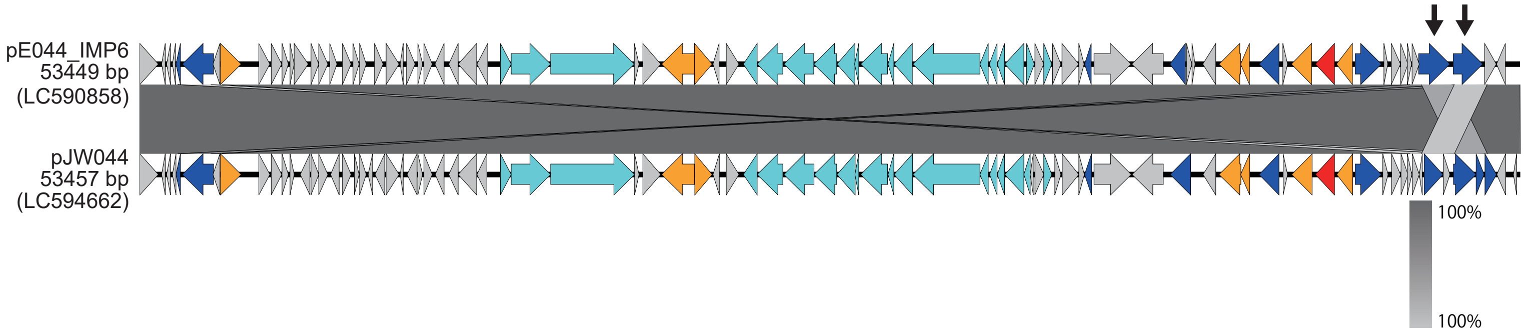

FIG S4 Comparison of the genome structures of plasmids pE044_IMP6 and pJW044. Plasmid pJW044 in *recA*-deficient transformant JW044, which was not multimerized, was compared with the original plasmid pE044_IMP6. The consecutive transposases likely targeted for homologous recombination leading to multimerization are indicated by arrows. The color code is as follows: red, carbapenem resistance gene; yellow, other antimicrobial resistance gene; light blue, conjugative transfer gene; blue, mobile element. Putative, hypothetical, or unknown genes are represented as grey arrows.
