# Supplementary material for: Enhanced Carbapenem Resistance through Multimerization of Plasmids Carrying Carbapenemase Genes
Source: mBio. 2021 Jun 22;12(3):e00186-21. doi: 10.1128/mBio.00186-21 (PMC8262910; doi:10.1128/mBio.00186-21)
Supplement: TEXT S1 [file mbio.00186-21-s0001.docx]

**Materials and Methods**

**Study isolates and Southern blotting**

*Escherichia coli* isolates E042, E044, E058, E059, E114, and E244, and *Klebsiella pneumoniae* isolate E188 were obtained during our previous carbapenem-resistant *Enterobacteriaceae* surveillance in Osaka (1). All isolates were subjected to S1-nuclease-digested pulsed-field gel electrophoresis (PFGE) followed by Southern blotting as previously described (2). Plugs containing these isolates were treated with proteinase K at 37 °C for 2 h followed by treatment with S1 nuclease (Takara Bio, Shiga, Japan) at 23 °C for 15 min. DNA fragments were separated using the CHEF-Mapper XA System (Bio-Rad, Hercules, USA) at 6.0 V/cm and 14 °C (switch time, 6.75.21.79 s) for 15 h. The DNA fragments were transferred to a nylon membrane using a ROYAL GENIE BLOTTER (Idea Scientific Company, Minneapolis, MN, USA) at 12 V for 1 h, hybridized with a digoxigenin-labeled probe (Roche, Basel, Switzerland) specific for the *bla*_IMP-6_ gene, and detected with CDP-Star Chemiluminescent Substrate (GE Healthcare Life Sciences, Chicago, IL, USA).

**Whole-genome sequencing and genome analysis**

Genomic DNA of *E. coli* isolate E044 and *E. coli* strain JW044 was extracted using the DNeasy PowerSoil Kit (Qiagen, Hilden, Germany) for both short- and long- read sequencing. Short-read sequencing was conducted on an Illumina MiSeq using the KAPA HyperPlus Library Preparation Kit (Kapa Biosystems, Basel, Switzerland) and long-read sequencing was conducted on a Nanopore GridION sequencer (Oxford Nanopore Technologies, UK) using the SQK-LSK109 1D Ligation Sequencing Kit and the EXP-NBD103 Native Barcoding Kit. The reads of isolate E044 were assembled and polished using Unicycler (3). The long reads of strain JW044 were assembled by Flye (4). Contigs were polished with BWA (5) and Pilon (6), using the short reads. The ResFinder (7) database was used to identify antimicrobial resistance genes and a detailed analysis of the insertion sequence was performed using ISfinder (8). The sequence annotated with RASTtk (9) was compared with that of plasmid pE188_IMP6, using Easyfig (10).

To analyze the genome structure of DNA fragments separated by S1-PFGE analysis, the DNA fragments of E044 with lengths of 50, 100, 150, and 200 kbp were extracted from the agarose using MagExtractor-PCR & Gel Clean up- (Toyobo Life Science, Osaka, Japan). Each DNA fragment was subjected to whole-genome sequencing analysis, using Illumina MiSeq. The reads were compared with the sequences of plasmid pE044_IMP6 and the chromosome of isolate E044, using CLC Genomics Workbench ver. 11 (Qiagen).

**Ultra-long read sequencing of pE044_IMP6 using MinION**

The library preparation protocol was partially modified from a previously described method (11). In the fragmentation step, the 1000 ng of genomic DNA of E044 strain was prepared using RB01 of fragmentation mix as previously described. The 7.5 µL of 400 ng genomic DNA was also fragmented with 2.5 µL of fragmentation mix (RB02) on at 30 °C for 1 min followed by 80 °C for 1 min on a thermocycler. After pooling 15 µL of fragmented RB01 and 5 µL of RB02, the following steps were performed as previously described, and sequenced using MinION sequencer. The sequence reads were mapped to the sequence of pE044_IMP6 using minimap2 (11). The mapped reads were compared with pE044_IMP6 using nucmer v. 4.0.0 (11) to calculate ratio mapped to pE044_IMP6.

**Conjugation and transformation**

Bacterial conjugation was performed using *E. coli* isolate E044 as a donor and the sodium azide-resistant *E. coli* strain TUM3456 (12) as a recipient. After mixing overnight cultures of donor and recipient at a 1:10 volumetric ratio, the mixture (10 μL) was incubated on lysogeny broth (LB) agar at 37 °C overnight. Transconjugants were selected on LB agar containing meropenem (0.125 μg/mL) and sodium azide (150 μg/mL). *bla*_IMP-6_ carriage was confirmed by PCR.

Plasmids were extracted from overnight culture of *K. pneumoniae* isolate E188 using the Plasmid Miniprep Kit (Qiagen). The extracted plasmid was electroporated into *E. coli* strain ME9062 and *recA* mutant JW2669-KC [NBRP (NIG, Japan): *E. coli*] using a Gene Pulser Xcell System (Bio-Rad, Hercules, USA). After incubation in S.O.C. Medium (Invitrogen, Waltham, USA) for 2 h, transformants were selected on LB agar containing 2 μg/mL cefotaxime, and *bla*_IMP-6_ carriage was confirmed by PCR. The transformants were indicated as ME188 and JW188, respectively.

The bacterial conjugation was performed using *E. coli* isolate E044 as a donor with functional *lacZ*, and *E. coli* strain ME9062 and *recA* mutant JW2669-KC as recipients with non-functional *lacZ*. After mixing the overnight cultures of donor and recipient at a 1:10 volumetric ratio, the mixture (10 μL) was incubated on LB agar overnight at 37 °C. White colonies were selected as transconjugants grown on LB agar containing 1 μg/mL cefotaxime, 40 μg/mL 5-bromo-4-chloro-3-indolyl-β-D-galactopyranoside, and 23 μg/mL Isopropyl β-D1-thiogalactopyranoside. *bla*_IMP-6_ carriage was confirmed by PCR, and these transconjugants were indicated as ME044 and JW044.

The plasmid of transconjugant JW044 was further conjugated into *E. coli* strain TUM3456 as mentioned above. The transformant was selected on LB agar supplemented with meropenem (0.125 μg/mL), sodium azide (150 μg/mL), and ZnSO4 (70 μg/mL), indicated as ME-JW044. *bla*_IMP-6_ carriage was confirmed by PCR, and the blue color of the colony of lactose-fermenting transconjugant were confirmed on LB agar supplemented with 5-bromo-4-chloro-3-indolyl-β-D-galactoside (40 μg/mL) and isopropyl-β-D-thiogalactopyranoside (23 μg/mL).

The transconjugants and transformants were subjected to S1-PFGE and Southern blot hybridization using a *bla*_IMP-6_ probe, following the same procedure as that used for the study isolates.

**Minimum inhibitory concentrations (MICs) of meropenem and measurement of *bla*_IMP-6_ copy numbers per cell**

The MICs of meropenem for transformants ME188, JW188, ME044, and JW044 were measured by the broth microdilution method in triplicate, according to the Clinical and Laboratory Standards Institute document M100-S28 (13).

To analyze *bla*_IMP-6_ copy numbers, DNA was extracted from an overnight culture in LB broth supplemented with meropenem (0.25 µg/mL), using the DNA Mini Kit (Qiagen). Using qPCR, *bla*_IMP-6_ copy numbers on plasmids were compared with the copy numbers of *rrsA*, encoding 16S ribosomal RNA on chromosome. qPCRs were carried out as previously described (2) using THUNDERBIRD SYBR qPCR Mix (Toyobo Life Science) on a LightCycler 96 System (Roche Life Science, Penzberg, Germany). qPCR analysis was performed using data from repeated experiments (n = 4) and the bla_IMP-6_ copy number per cell was calculated based on Ct values, using the comparative Ct method (14).

**Analysis of transcription of *bla*_IMP-6_**

*E. coli* transformants ME188, JW188, ME044, and JW044 were incubated in LB broth supplemented with meropenem (0.25 µg/mL) overnight, the cultures (each 50 µL) were incubated in LB broth (3 mL), and cultured further until the optical density at 600 nm reached 0.3–0.4. Total RNA was extracted using the RNeasy Mini Kit (Qiagen). RNA was treated with ReverTra Ace qPCR RT Master Mix with gDNA Remover (Toyobo Life Science) to remove contaminating DNA and reverse-transcribe the RNA into cDNA. For quality control, DNase-treated RNA that had not been reverse-transcribed was subjected to a DNA-contamination test by qPCR. Transcription of *bla*_IMP-6_ was calculated by the comparative Ct method, using *rrsA* as an endogenous control for normalization. The qPCR analysis was repeated five times.

**Measurement of MICs of meropenem and *bla*_IMP-6_ copy numbers per cell after meropenem treatment**

Each colony from overnight cultures of transformants ME188 and ME044 on Mueller Hinton II agar was incubated in Mueller Hinton II broth at 37 °C for 12 h. Each culture (3 µL) was inoculated in Mueller Hinton II broth (3 mL), or in Mueller Hinton II broth supplemented with meropenem (0.25 µg/mL), and incubated at 37 °C overnight. Using the cultures, the MICs of meropenem and *bla*_IMP-6_ copy numbers per cell were measured.

To analyze *bla*_IMP-6_ copy numbers, DNA was extracted from the culture using the DNA Mini Kit (Qiagen). Using qPCR, *bla*_IMP-6_ copy numbers were calculated by the comparative Ct method with *rrsA* as an endogenous control for normalization.

To analyze the MIC of meropenem, the cultures were washed twice with phosphate-buffered saline (PBS) and then suspended in PBS at a turbidity equivalent to a 1.0 McFarland standard. The suspensions were diluted in Mueller Hinton II broth at a ratio of 1:600, supplemented with meropenem in serial two-fold dilutions (1–256 µg/mL). After incubation in a 96-well plate at 37 °C for 18 h, MICs were determined according to the turbidity of the wells. All procedures were repeated nine times.

In addition, MICs of meropenem were compared with ETEST**®** (bioMérieux, Marcy l’Etoile, France), using cultures suspended in PBS at a turbidity equivalent to a 0.5 McFarland standard after washing twice with PBS.

**References**

1. Yamamoto N, Asada R, Kawahara R, Hagiya H, Akeda Y, Shanmugakani RK, Yoshida H, Yukawa S, Yamamoto K, Takayama Y, Ohnishi H, Taniguchi T, Matsuoka T, Matsunami K, Nishi I, Kase T, Hamada S, Tomono K. 2017. Prevalence of, and risk factors for, carriage of carbapenem-resistant *Enterobacteriaceae* among hospitalized patients in Japan. J Hosp Infect 97:212–217.

2. Abe R, Akeda Y, Sugawara Y, Takeuchi D, Matsumoto Y, Motooka D, Yamamoto N, Kawahara R, Tomono K, Fujino Y, and SH. 2020.Characterization of the plasmidome encoding carbapenemase and mechanisms for dissemination of carbapenem-resistant *Enterobacteriaceae*. mSystems 5:e00759-20.

3. Wick RR, Judd LM, Gorrie CL, Holt KE. 2017. Unicycler: Resolving bacterial genome assemblies from short and long sequencing reads. PLoS Comput Biol 13:e1005595.

4. Kolmogorov M, Yuan J, Lin Y, Pevzner PA. 2019. Assembly of long, error-prone reads using repeat graphs. Nat Biotechnol 37:540–546.

5. Li H. 2013. Aligning sequence reads, clone sequences and assembly contigs with BWA-MEM. arXiv preprint arXiv:1303.3997.

6. Walker BJ, Abeel T, Shea T, Priest M, Abouelliel A, Sakthikumar S, Cuomo CA, Zeng Q, Wortman J, Young SK, Earl AM. 2014. Pilon: an integrated tool for comprehensive microbial variant detection and genome assembly improvement. PLoS One 9:e112963.

7. Zankari E, Hasman H, Cosentino S, Vestergaard M, Rasmussen S, Lund O, Aarestrup FM, Larsen MV. 2012. Identification of acquired antimicrobial resistance genes. J Antimicrob Chemother 67:2640–2644.

8. Siguier P, Perochon J, Lestrade L, Mahillon J, Chandler M. 2006. ISfinder: the reference centre for bacterial insertion sequences. Nucleic Acids Res 34:D32-6.

9. Brettin T, Davis JJ, Disz T, Edwards RA, Gerdes S, Olsen GJ, Olson R, Overbeek R, Parrello B, Pusch GD, Shukla M, Thomason JA 3rd, Stevens R, Vonstein V, Wattam AR, Xia F. 2015. RASTtk: a modular and extensible implementation of the RAST algorithm for building custom annotation pipelines and annotating batches of genomes. Sci Rep 5:8365.

10. Sullivan MJ, Petty NK, Beatson SA. 2011. Easyfig: a genome comparison visualizer. Bioinformatics 27:1009–1010.

11. Jain M, Koren S, Miga KH, Quick J, Rand AC, Sasani TA, Tyson JR, Beggs AD, Dilthey AT, Fiddes IT, Malla S, Marriott H, Nieto T, O’Grady J, Olsen HE, Pedersen BS, Rhie A, Richardson H, Quinlan AR, Snutch TP, Tee L, Paten B, Phillippy AM, Simpson JT, Loman NJ, Loose M. 2018. Nanopore sequencing and assembly of a human genome with ultra-long reads. Nat Biotechnol 36:338–345.

12. Ma L, Ishii Y, Ishiguro M, Matsuzawa H, Yamaguchi K. 1998. Cloning and sequencing of the gene encoding Toho-2, a class A beta-lactamase preferentially inhibited by tazobactam. Antimicrob Agents Chemother 42:1181–1186.

13. Clinical and Laboratory Standards Institute. M100 Performance Standards for Antimicrobial Susceptibility Testing, 28th edition. 2018. <http://file.qums.ac.ir/repository/mmrc/CLSI-2018-M100-S28.pdf>.

14. Schmittgen TD, Livak KJ. 2008. Analyzing real-time PCR data by the comparative C(T) method. Nat Protoc 3:1101–1108.
