# Supplementary figures and images for: Enhanced Carbapenem Resistance through Multimerization of Plasmids Carrying Carbapenemase Genes
Source: mBio. 2021 Jun 22;12(3):e00186-21. doi: 10.1128/mBio.00186-21 (PMC8262910; doi:10.1128/mBio.00186-21)

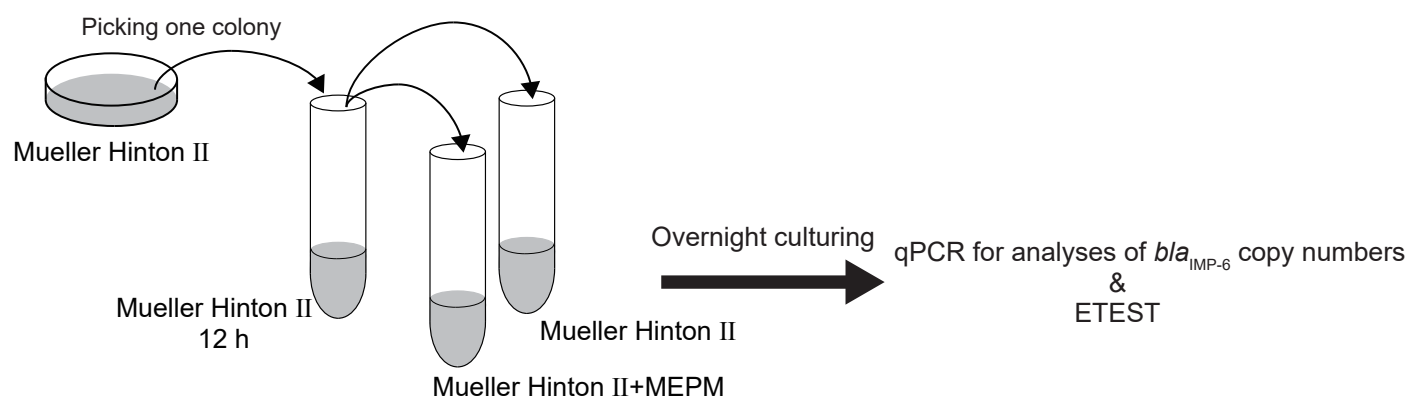

**FIG S5. Schematic procedure of meropenem pre-exposure.**

Supplement: FIG S5 [file mbio.00186-21-sf005.pdf]
