# Supplementary material for: Enhanced Carbapenem Resistance through Multimerization of Plasmids Carrying Carbapenemase Genes
Source: mBio. 2021 Jun 22;12(3):e00186-21. doi: 10.1128/mBio.00186-21 (PMC8262910; doi:10.1128/mBio.00186-21)
Supplement: FIG S6 [file mbio.00186-21-sf006.pdf]

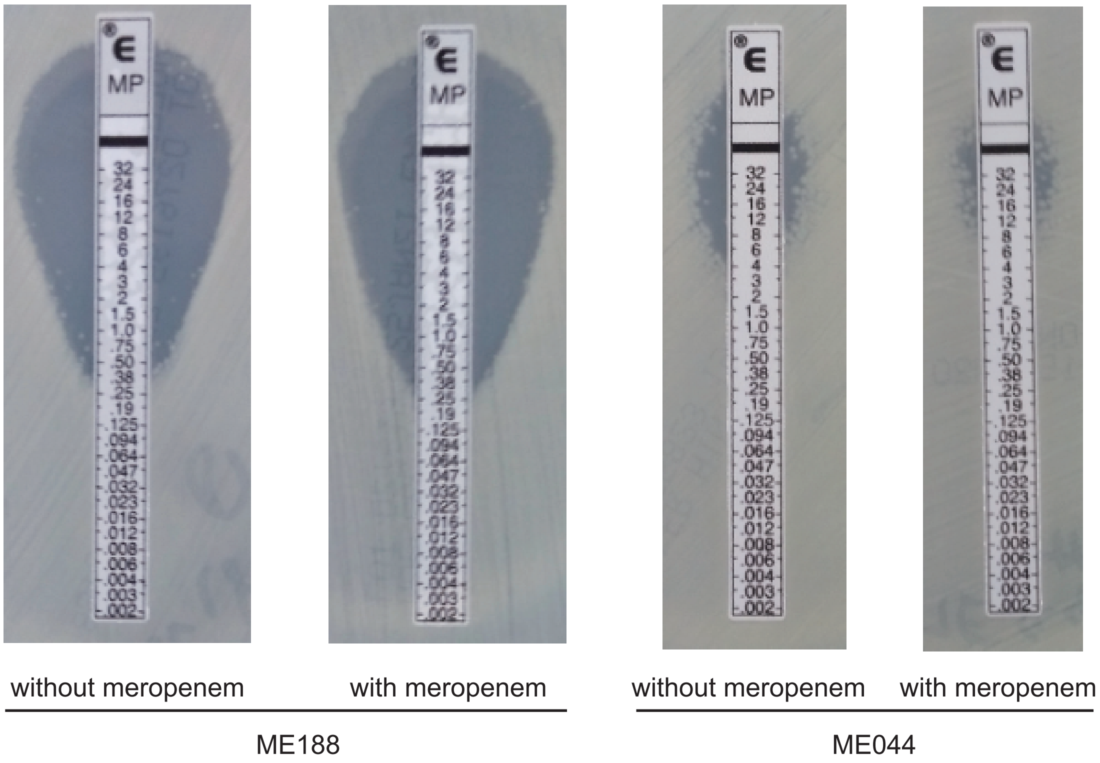

FIG S6 Emergence of enhanced meropenem resistant clone through meropenem exposure. Meropenem MICs of isolates ME188 and ME044 with or without meropenem exposure were measured by ETEST.
